# Evaluating Aggregation Membership and Copulatory Success in the Stink Bug, *Euschistus conspersus*, using Field and Laboratory Experiments

**DOI:** 10.1673/031.011.0102

**Published:** 2011-01-12

**Authors:** Christian H. Krupke, Vincent P. Jones, Jay F. Brunner

**Affiliations:** ^1^ Department of Entomology, Purdue University, 901 West State St., West Lafayette, IN 47907-2089; ^2^ Department of Entomology, Washington State University, Tree Fruit Research and Extension Center, 1100 North Western Ave., Wenatchee, WA 98801

**Keywords:** behavior, mate choice, size

## Abstract

The aggregation and mating behavior of the stink bug, *Euschistus conspersus* Uhler (Hemiptera: Pentatomidae) was investigated in a series of field and laboratory experiments. Marking of *E. conspersus* mating in aggregations in the field demonstrated that both sexes mate multiple times within aggregations on successive nights and with different partners, although ≈ 20% of the individuals of both sexes returned to aggregations but did not mate. Further analysis of mating patterns in caged aggregations revealed that heavy males and light females mated more frequently than their respective counterparts. Data are interpreted in terms of elucidating the function of benefits of multiple mating within aggregations for males and females.

## Introduction

The stink bug, *Euschistus conspersus* Uhler (Heteroptera: Pentatomidae) is endemic to western North America (Beers et al. 1993). The mating period of this insect occurs once annually, in the spring through early summer. Adults mate a maximum of once daily, in aggregations of 5–35 individuals (Alcock 1971; Krupke et al. 2001) with matings lasting from 3–35 hours (Hunter and Leigh 1965). Generally, aggregations are assumed to benefit individuals within them by enabling the responders to compare quality of potential mates and by improved rates of predator detection and enhanced defense (Allee 1931; Thornhill and Alcock 1983). However, in the case of *E. conspersus* there may be significant costs associated with the production of, and/or the response to, the aggregation pheromone. The major component of this pheromone, methyl (*E*)2 (*Z*)4-decadienoate, is used as a host-location kairomone for *Gymnoclytia occidentalis* Townsend (Diptera: Tachinidae), a commonly occurring parasitoid attacking *E. conspersus* in the area of this study (Krupke and Brunner 2003). Heightened levels of parasitism in aggregations have been shown with another stink bug species, *Nezara viridula* (Linnaeus), and its parasitoid, *Trichopoda pennipes* (F.) (Diptera: Tachinidae) (Mitchell and Mau 1971; Harris and Todd 1980), and it was hypothesized that this may hold true for *E. conspersus* as well. It is reasonable to expect that females, who do not produce pheromone, will incur a higher parasitism risk while in aggregations containing pheromone-producing males (Wertheim et al. 2003). However, the sex ratios and memberships of these aggregations have never been empirically investigated.

To clarify these aspects of aggregation formation in this species, field experiments were performed to determine whether insects forming aggregations did so over successive nights and if there were sex-specific differences among repeat aggregators. To generate detailed information about the characteristics (i.e. weight and size) of the aggregators, laboratory-generated aggregations were used to examine one possible factor that may cause some individuals to have greater mating success than others, the relative sizes of insects. Our *a priori* hypotheses were that there would be sex-specific differences in insects that returned to aggregations, and that size of insects would predict mating success in laboratory aggregations as shown in studies of other stink bug species (McLain 1980; Himuro et al. 2006).

## Materials and Methods

### Field aggregation labeling

To document membership in natural aggregations one of the common host plants in the study area, common mullein, *Verbascum thapsus* L. (Lamiales: Scrophulariaceae), was used. Twenty plants were randomly chosen at two sites bordering commercial orchards in Chelan Co., WA, with the constraint that plants were never less than 40 m apart. At each site, the plants were spread along transects ∼1000 m long. Two individuals made observations simultaneously. Plants were surveyed hourly during the peak period of mating initiation, which occurs from 19:00 to 23:00 (Krupke et al. 2006), using a red, darkroom safelight (Model E.26, Kodak Corp. www.kodak.com) after sunset to minimize disturbance to the insects. At each interval, the total number of *E. conspersus* present on each plant and the total number of pairs found mating was recorded. Any individuals found mating were carefully marked on the middle of the scutellum of each individual with a small (∼ 1 mm diameter) dab of nail polish (Super Shine Naturistics®, Del Laboratories Inc., www.dellabs.com). Insects were remarked each time they were found mating, with different colors used on each night of the study. The experiment was conducted during the period of 9–13 June 2004. Numbers of marked males and marked females found remating in aggregations on subsequent nights were analyzed using a heterogeneity chisquare test to determine whether either sex was significantly more likely to re-mate in the aggregation on a given night.

### Collection and rearing of insects

Reproductively immature adult *E. conspersus* were field-collected from common mullein at areas surrounding orchards in Chelan and Douglas counties in Washington State. Collection began in late March and was completed by mid-April 2004, closely following emergence of the insects from overwintering sites. All insects were maintained in an unheated greenhouse that received only ambient light in order to preserve natural diurnal behavioral cycles. Males and females were held separately in screen cages measuring 1 m long by 1.5 m tall and 1 m deep. Each cage contained 4 potted mullein plants that the insects used as perching substrates and food and water sources, supplemented by organic green beans, *Phaseolus vulgaris* L. (Fabales: Fabaceae) and small pieces of cardboard with several raw organic sunflower, *Helianthus annuus* L (Asterales: Asteraceae) and peanut, *Arachis hypogaea* L. (Fabales: Fabaceae) seeds glued to their surface using white Elmer's glue. All food sources were replaced twice weekly.

### Mating in caged aggregations

Twenty virgin *E. conspersus* of each sex were placed inside each of three 0.5 m × 0.5 m × 1.0 m screen cages containing a potted mullein plant. Individual insects were identified by a small nail-polish mark on the scutellum, then weighed using a digital scale accurate to 0.1 milligrams (Ohaus Inc., Model 200s, www.ohaus.com) and measured before being placed inside the cage. The pronotal width of each insect to the nearest 0.01 mm was taken using Max-Cal™ digital vernier calipers (Foster & NSK Inc., Japan). The nail polish mark was used to identify and track each insect individually throughout the experiment. All insects used in the aggregation cages were also categorized as “heavy” or “light” depending on whether their weights were above or below the median weight of the entire group for each sex. The 120 insects used in the experiment were thus divided into four groups of 30 individuals each: heavy males, heavy females, light males, and light females. Ten individuals from each group were placed into each of the three cages at approximately 9:00. Cages were held in the greenhouse and received only natural light and were examined at hourly intervals, using a red safelight during hours of darkness throughout the mating period, which lasted from 19:00 until 6:00. When mating pairs were found, they were gently removed from the cage and placed inside individual 946 ml plastic cups (Solo Cup Co. www.solocup.com) containing live mullein leaves. This allowed the insects to complete mating and facilitated positive identification and recording of mating partners the following morning. All insects were reinserted into the screen cage following the end of mating each morning. This experiment was conducted in three cages simultaneously and data were collected over nine consecutive nights. Data from all cages were combined for analysis, unless otherwise stated.

### Statistical analysis

Linear regression was performed using statistical software (SPSS Inc. 2006) to determine whether pronotal width or weight were predictors of the number of matings in the laboratory aggregations. Bivariate correlation analysis was also performed on these data to determine whether pronotal width was a significant predictor of weight in each sex.

## Results

### Field aggregation labeling

**Figure 1.**
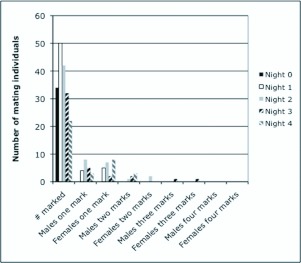
Total numbers of mating male and female *Euschistus conspersus* marked and found re-mating in field aggregations over a four-night period. Heterogeneity χ^2^ analysis found no significant differences in numbers of males and females. High quality figures are available online.

χ^2^ analysis revealed that marked males and females were equally likely to return to mate in field aggregations on subsequent nights (χ^2^ = 0.06, df = 1, P = 0.81) (Figure 1). Interestingly, no individuals were found remating with the same partner (i.e. a partner with the same-color mark) over the observation period. The non-mating returning individuals represented 21.2% and 20.9% overall of the marked males and females returning to plants, respectively. The mean (SE) percentage of marked individuals with at least one mark found re-mating over the 4night period following the initial marking were as follows: Night 1–18.8 (5.4), Night 2 - 50.0 (8.6), Night 3–23.1 (7.5), Night 4 – 63.8 (11.1). Approximately 70% of both males and females mated once and 20% mated twice over the five-night observation period.

### Mating in caged aggregations

A total of 292 matings were recorded in the caged aggregations over the 9 nights of the experiment. Among insects that mated at least once, the mean (SE) number of matings over the 9-night period was 5.28 (0.36) mating for females and 4.98 (0.28) matings for males. In both sexes weight and pronotal width exhibited a significant correlation: Pearson's correlation coefficient females: 0.661, P < 0.01; Pearson's correlation coefficient males: 0.632, P < 0.01.

**Figures 2.**
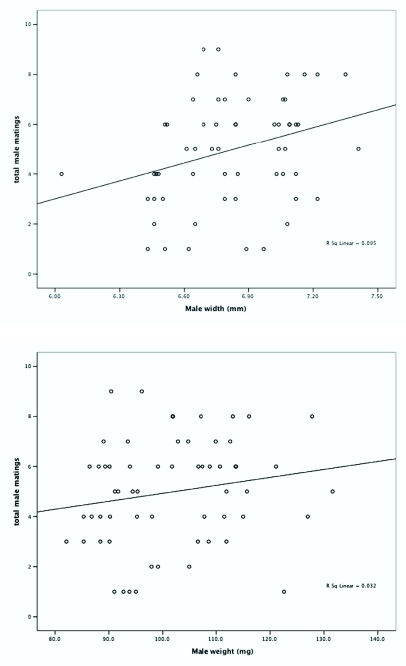
Total male matings predicted by (a) male width and (b) male weight using logistic regression analysis. Individual data points are shown as (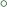). High quality figures are available online.

**Figures 3.**
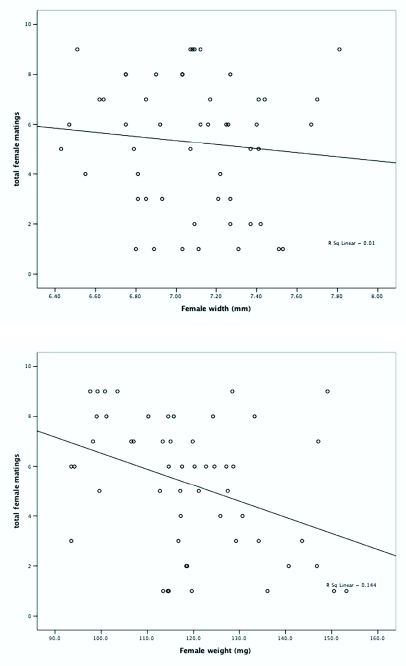
Total female matings predicted by (a) female width and (b) female weight using logistic regression analysis. Individual data points are shown as (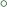). High quality figures are available online.

Linear regression analysis revealed that female weight and male pronotal width were significant predictors of mating success, and lighter females mated more often than heavy ones, and males with wider pronotums mated more than their narrower counterparts: female weight (*F* = 8.608; df = 1, 51; *P* < 0.005; r^2^ = 0.144), male width (*F* = 6.064; df = 1, 58; *P* = 0.017; r^2^ = 0.095). Female width (*F* = 0.528; df = 1, 51; *P* = 0.471; r^2^ = 0.010) and male weight (*F* = 1.910; df = 1, 58; *P* < 0.172; r^2^ = 0.032) were not significant predictors of mating success (Figure 2 and Figure 3). A total of 41 out of 60 insects of each sex mated on the first night of the experiment. Of the remaining 19 individuals of each sex, one male and five females did not mate throughout the experiment.

## Discussion

The experiments with field aggregations demonstrated that *E. conspersus* aggregations re-form on the same mullein plant each night. The methodology did not enable distinguishing whether insects moved from one of our plants to another on successive nights. That is, labels were given only to color-code insects mating on a given night of the study, not for specific plants. However, the presence of equal proportions of marked males and females found mating in aggregations on subsequent nights was somewhat surprising - it may have been expected that males would display a shorter refractory period because male bugs that mate with multiple females benefit by potentially siring additional offspring and perhaps by mate-guarding (McLain 1985). Conversely, for females there may be heightened parasitism risk associated with membership in aggregations (Krupke and Brunner 2003), suggesting that females should mate less often than males. These experiments demonstrated that this is not the case, leaving open the possibility that females are gaining important direct (e.g. nutritional — discussed further below) or indirect benefits by re-mating that outweigh the possible increased risk of parasitism. Indirect benefits may include increasing genetic variability of progeny females may be more likely to mate when provided access to many possible mates at a single location. This has been found in studies of other stink bugs - females of *Megacopta punctatissima* (Montandon) were shown to shown to accept males courting within aggregations more often than solitary courters (Hibino 1986). Males may have to limit their mating frequency because of the time and energy costs associated with mating (Krupke et al. 2008).

An interesting result of the experiments with caged aggregations is the observation that large males and light females mated with greater frequency than their respective counterparts. The observation that large males mate more frequently than light ones may be due to more aggressive courting and/or endurance rivalry (Andersson 1994), where large males are better able to remain in the aggregation and lay claim to prime areas on the plant. This may be important to faciliate substrate-based transmission of acoustical mating signals (Ota and Cokl 1991), used extensively by *E. conspersus* during courtship (McBrien and Millar 2003). Large males have been shown to secure more mates than their smaller counterparts in studies of other aggregating stink bug species as well, and small male mating success can be increased significantly when large competitors are excluded in laboratory studies (Himuro et al. 2006).

Light females may mate more frequently because they benefit more than their heavier counterparts by obtaining direct benefits (i.e. materials transferred by males in addition to sperm) during mating. This possibility is supported by a previous study (Krupke et al. 2008), which found that females that mated lived significantly longer than those that did not, when both were denied access to food and water. These benefits from mating may present an important incentive for females to return to aggregations, particularly when resources are scarce. Alternatively, light females may mate more simply because they are preferred by males — the methodology used did not allow determination as to whether this was the case, so this possibility cannot be ruled out.

Our studies of field aggregations revealed that males and females are equally likely to mate on successive nights. This, along with the equal frequencies of mating among males and females observed in caged aggregations, suggests that males and females of this species have an equivalent refractory period before remating, meaning that the operational sex ratio should remain approximately equal over the mating season. Aggregations may increase male mating frequency while facilitating female choice simply by providing access to many possible mates at a single location.
